# To be funny or not to be funny: Gender differences in student perceptions of instructor humor in college science courses

**DOI:** 10.1371/journal.pone.0201258

**Published:** 2018-08-15

**Authors:** Katelyn M. Cooper, Taija Hendrix, Michelle D. Stephens, Jacqueline M. Cala, Kali Mahrer, Anna Krieg, Ashley C. M. Agloro, Giovani V. Badini, M. Elizabeth Barnes, Bradley Eledge, Roxann Jones, Edmond C. Lemon, Nicholas C. Massimo, Annette Martin, Thomas Ruberto, Kailey Simonson, Emily A. Webb, Joseph Weaver, Yi Zheng, Sara E. Brownell

**Affiliations:** 1 School of Life Sciences, Biology Education Research Lab, Arizona State University, Tempe, Arizona, United States of America; 2 School of Life Sciences, Biology Education Research Class, BIO 494/598 Arizona State University, Tempe, Arizona, United States of America; 3 Mary Lou Fulton Teachers College, Arizona State University, Tempe, Arizona, United States of America; IUMPA - Universitat Politecnica de Valencia, SPAIN

## Abstract

For over 50 years instructor humor has been recognized as a way to positively impact student cognitive and affective learning. However, no study has explored humor exclusively in the context of college science courses, which have the reputation of being difficult and boring. The majority of studies that explore humor have assumed that students perceive instructor humor to be funny, yet students likely perceive some instructor humor as unfunny or offensive. Further, evidence suggests that women perceive certain subjects to be more offensive than men, yet we do not know what impact this may have on the experience of women in the classroom. To address these gaps in the literature, we surveyed students across 25 different college science courses about their perceptions of instructor humor in college science classes, which yielded 1637 student responses. Open-coding methods were used to analyze student responses to a question about why students appreciate humor. Multinomial regression was used to identify whether there are gender differences in the extent to which funny, unfunny, and offensive humor influenced student attention to course content, instructor relatability, and student sense of belonging. Logistic regression was used to examine gender differences in what subjects students find funny and offensive when joked about by college science instructors. Nearly 99% of students reported that they appreciate instructor humor and reported that it positively changes the classroom atmosphere, improves student experiences during class, and enhances the student-instructor relationship. We found that funny humor tends to increase student attention to course content, instructor relatability, and student sense of belonging. Conversely, offensive humor tends to decrease instructor relatability and student sense of belonging. Lastly, we identified subjects that males were more likely to find funny and females were more likely to find offensive if a college science instructor were to joke about them.

## Introduction

Students often perceive science courses to be difficult, competitive, and boring and science instructors have been stereotyped as dull and described as unapproachable [1–5]. Although these perceptions can be difficult to alter, one classroom practice that has the potential to positively change undergraduates’ perceptions of science instructors and science classrooms is instructor use of humor.

Humor is commonly defined as the quality of being amusing or funny [6]. Although humor is subjective and it is often difficult to describe why something is funny, the research literature on humor suggests that what is often humorous is what is unexpected from the norm [7–9]. People use humor for many different reasons [9]; humor can be used to increase group cohesion [7,9–11] to relieve stress [9,12], or to assert superiority [9,12].

College instructors have been shown to regularly use humor during class [13–15]. One study that sampled from 70 college courses across different academic disciplines found that 80% of instructors used humor at least once during a randomly selected 50-minute lecture [13]. For over 50 years, instructor humor has been recognized as a way to positively impact student cognitive and affective learning [9,16–21]. For example, studies have shown that humor in the college classroom is positively related to student sense of community in the classroom [16], student attention during class [16–18], student comfort asking questions of the instructor [19], student participation in class [20], and student motivation to attend class [19]. Further, students self-report that humor improves their learning [19,21], although research results conflict about whether humor actually enhances student learning. Some studies have found no relationship between humor and student learning [22,23], while other studies have found that humor has a positive effect on student learning [9,24–26]. Notably, to our knowledge, no studies have explored the benefits of instructor humor specifically in the context of college science courses, which are often perceived as difficult and competitive [1–5].

The majority of studies that explore the effect of instructor humor on students have assumed that students perceived the humor to be funny, yet it is likely that students experience instructor humor that they perceive as unfunny or may even consider to be offensive. In fact, one study surveyed 124 students across three college classes about instructor use of humor and when students were asked to report possible problems with using humor in class, 32% of students identified that humor has the potential to be offensive [16]. Further, students in an introductory communications course generated 513 examples of instructor humor that they considered to be inappropriate, many of which were disparaging to students [24]. Even though there is evidence for what students perceive to be offensive or inappropriate forms of humor, to our knowledge no studies have explored how instructor use of offensive humor may influence students’ experiences in the science classroom.

Further, there is some evidence that female students perceive certain subjects to be more offensive than male students do. Studies have shown that female students are less tolerant of jokes about male or female stereotypes that are crude or profane [25] and female students are less likely than male students to enjoy sexual humor [26]. We do not know if women are more offended by topics of jokes that may be used by instructors in college science classrooms, nor do we know what impact offensive humor may have on the experience of women in science classes.

We do know however, that undergraduate women in college science courses have reported lower sense of belonging [27–29], lower confidence [30,31], and lower perception of their academic abilities compared to their male counterparts [30–33]. Further, evidence suggests that women may be less engaged in science classes [34]; specifically, studies show that, compared to males, females have a lower preference for being a leader in small group discussion [35] and do not participate as much in whole class discussion in college science courses [36]. Studies have also shown that female science, technology, engineering, and math (STEM) majors report significantly lower respect and recognition from STEM instructors [30] and are less likely to perceive that instructors know their name [37]. Notably, many of these gender disparities have been found across undergraduate science courses, even in disciplines such as biology where women make up 60% of undergraduate majors [30,31,38–40]. Could instructor use of humor be a factor negatively affecting the experience of women in college science courses?

In this manuscript, we set out to explore student perceptions of instructor use of humor in college science classrooms and whether there are any gender differences in how students perceive and are affected by instructor use of humor. The specific research questions of each study are as follows:

Study I: To what extent do students appreciate when instructors use humor in college science classes? Why do students appreciate when instructors use humor in college science classes?

Study II: How do instructors’ use of funny humor, unfunny humor, and offensive humor in college science courses affect student attention to course content, instructor relatability, and student sense of belonging to the course? Are there gender differences in the extent to which students report being affected by funny, unfunny, and offensive humor?

Study III: When instructors use humor in college science classes, what potentially humorous subjects are students likely to find funny? What potentially humorous subjects are students likely to find offensive? Are there potentially humorous subjects that male or female students are more likely to find funny or offensive?

## Methods and results

This study was done with an approved Arizona State University Institutional Review Board protocol #00005725.

This research project was conducted as part of a biology education course-based research experience (CRE) taught by KMC, MEB, and SEB in the spring semester of 2017. A CRE is a course where students engage in novel, broadly relevant research [41,42]. This course was backward designed with the goal of teaching students about biology education research by exploring a research question that could result in publication [43]. Sixteen students were enrolled in the semester-long 3 unit course. The instructors of the course and the student researchers collectively were responsible for developing the research questions, collecting data, analyzing data, interpreting data, and communicating the findings. See Cooper and Brownell (under review [44]) for a more detailed description of the structure and organization of this CRE.

### Humor survey development and distribution

No previously developed survey existed to explore student perceptions of instructor use of humor in college science classrooms, so we designed a survey based on our specific research questions and the prior literature. We iteratively reviewed and modified the survey questions using a set of criteria that we developed to assess the appropriateness of each question (e.g. Is the question grammatically correct? Is the meaning and interpretation of the question clear? Are the question answer choices unambiguous in meaning?) [45]. Seventeen researchers reviewed the survey and evaluated the appropriateness of survey questions based on the criteria [45]. The researchers provided written feedback about each question and the survey was revised. Next, three of the researchers (GVB, EAW, RJ) conducted a series of think-aloud interviews with a total of eight undergraduate biology students to establish cognitive validity of the humor survey by ensuring that students understood what each question was asking. The survey was iteratively revised after each think-aloud interview [46]. Seventeen of the researchers completed the revised humor survey and again evaluated each question using the criteria for assessing survey questions. Once again, the survey was revised based on their feedback. Finally, the humor survey was piloted with one biology education post-doc, three biology education graduate students, and three undergraduate biology students, none of whom were involved with the project. The survey was revised a final time based on their feedback. Thus, the humor survey was iteratively revised a total of 11 times with 49 instances of individual feedback. Please see the Supplemental Information (S1 File) for questions from the final humor survey.

Data were collected from a large Research 1 institution in the Southwest United States. We recruited instructors to deploy the survey in their science classes. Instructors offered students a small amount of extra-credit for completing the ~15 minute survey. In cases where an instructor was not able to offer extra-credit, students were offered a chance to win a $200 gift card for completing the survey.

The survey was deployed using the online platform Qualtrics in 25 different undergraduate science classes, including courses in biology, chemistry, physics, and environmental science. Once instructors deployed the survey, students were given approximately one week to complete it. The Institutional Review Board at the institution where this study was conducted requires that students consent at the beginning of the survey to have their data used for research purposes. Once all data were collected, student names were immediately removed from survey responses and replaced with random identifiers. Two researchers (JMC and KM) cleaned the data by removing all entries from students who did not consent to participate in the study and from students who did not finish completing the survey. The researchers also deleted any duplicate responses from students who completed the survey more than once, leaving a complete set of 1637 student responses. Demographics of the students who consented to having their data included in the study are shown in **Table 1**.

**Table 1:** Demographics of students who completed the humor survey.

		% of Students (n = 1637)
Gender	Female	61.3%
	Male	37.0%
	Other	0.6%
	Decline to state	1.0%
Race/ethnicity	American Indian, Native American, or Alaskan Native	0.5%
	Asian	14.6%
	Black or African American	4.2%
	Hispanic or Latino or Spanish	12.5%
	Native Hawaiian or Other Pacific Islander	0.5%
	White/Caucasian	49.8%
	Multiple races	11.7%
	Other	3.4%
	Decline to state	2.7%
Age	18–22	86.3%
	23–27	8.4%
	28–32	1.3%
	33+	1.6%
	Decline to state	2.3%
Major	Biological Sciences major	57.5%
	Chemistry or Biochemistry major	12.2%
	Engineering major	9.3%
	Other major (e.g. Psychology, Computer Science, Business)	19.2%
	Decline to state	1.9%

This study was conducted at an institution in the United States and we recognize that humor is highly dependent on culture and thus, these findings may not be translatable to non-Western cultures [9,47].

### Study I: To what extent do students appreciate when instructors use humor in college science classes? Why do students appreciate when instructors use humor in college science classes?

#### Study I Methods

To determine the extent to which students appreciate when instructors use humor in college science classes, we analyzed the survey question “Please indicate the degree to which you agree with the following statement: I appreciate when instructors use humor in college science classrooms,” which students answered using a 6-point Likert-scale ranging from 1 = strongly disagree to 6 = strongly agree. We designed a 6-point Likert-scale because we wanted to know whether students either agreed or disagreed with the statement and our think-aloud interviews indicated that students were not neutral or ambivalent about the extent to which they appreciated instructor humor.

Students who strongly agreed or agreed that they appreciate when instructors use humor in college science classrooms were asked to explain their reasoning for why they appreciate when instructors use humor in college science classrooms. Four researchers (TH, ECL, AK, and TR) reviewed student responses to this open-ended question using inductive coding [48]. We probed why students appreciate instructors’ use of humor in undergraduate science courses without a specific hypothesis in mind because this question has never been explored in the context of undergraduate science courses. Thus, we did not want to bias our findings and we let themes emerge from the data [49]. Together, the researchers analyzed a subset of 500 student responses and developed a rubric to describe the most apparent themes. Two researchers (KMC and SEB) reviewed the rubric and 200 student responses to ensure that the rubric was representative of the most apparent themes. Then, using the rubric, the four researchers (TH, ECL, AK, and TR) individually analyzed 200 student responses using constant comparison methods [50]. They assigned each quote to a theme and constantly compared quotes to each other to ensure that each quote fit within the description of the theme that it was assigned to and to ensure that quotes were not different enough to warrant another category. A single student’s response could consist of multiple quotes. After individually coding 200 responses, the researchers compared codes and revised the rubric. This process was repeated until there was a consensus estimate of at least 70% among all four researchers. Once reaching a consensus estimate of 70%, the four researchers individually used the rubric to code every student response. Finally, the researchers compared their codes for every student response and came to consensus when they disagreed. See Supporting Information (S2 File) for a copy of the coding rubric.

#### Study I Results

**Fig 1:**
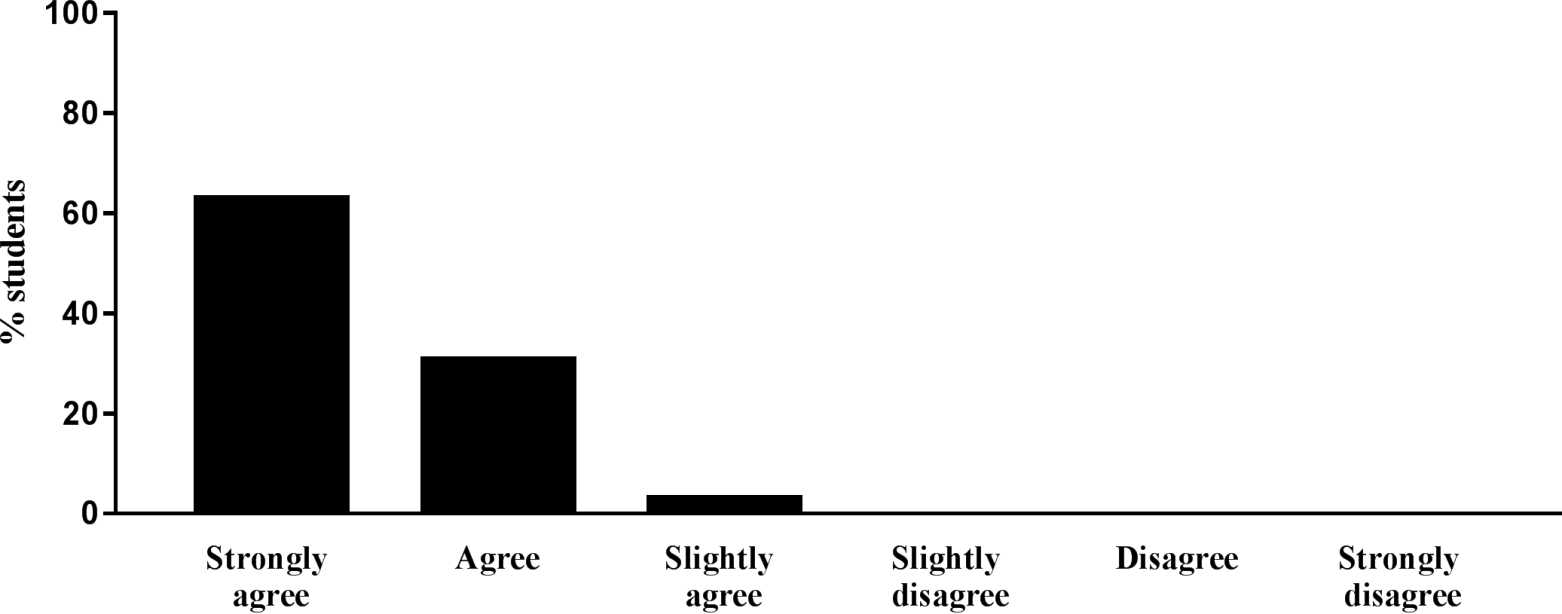
Student responses to the statement “I appreciate when instructors use humor in college science classrooms”.

The majority of students strongly agreed (63.7%), agreed (31.5%), or slightly agreed (3.7%) with the statement “I appreciate when instructors use humor in college science classrooms.” Very few students strongly disagreed (0.4%), disagreed (0.2%), or slightly disagreed (0.5%) with the statement. Collapsing the data, 98.8% of students agreed and only 1.2% of students disagreed that they appreciate when instructors use humor in college science classrooms (**Fig 1).**

Students who strongly agreed or agreed that they appreciate when instructors use humor in college science classrooms were asked why they appreciate when instructors use humor. The inductive coding analysis generated nine themes. Of the 1557 students who strongly agreed or agreed that they appreciated humor, 1541 students provided a response to the question and 1475 of the responses (94.7%) were able to be coded. The nine themes were grouped into three larger categories: (1) humor positively changes the classroom environment, (2) humor improves students’ experience in class, and (3) humor enhances the relationship between students and the instructor. Students were able to write as much as they wanted in response to this question and 1139 students (77.2% of students who provided responses that could be coded) reported more than one reason for why they appreciate when instructors use humor in college science classrooms. The average number of reasons that a student reported was 1.62. The percent of responses that fell into a particular category was calculated by dividing the number of responses by the number of students who provided a response that could be coded (n = 1475).

Students reported that they appreciate when instructors use humor in college science classes because it positively changes the classroom atmosphere (**Table 2**). Specifically, 49.4% of students appreciate science instructors’ use of humor because it makes class more interesting, fun, or exciting and makes the class feel less boring. Students (21.8%) also described how science classes can feel “dark” or “heavy” and when science instructors use humor, it lightens the mood of the class and creates a more comfortable and inviting environment. Further, students (7.8%) acknowledged that science content can be difficult and when instructors use humor, it gives students a break from the hard content and allows for more time to process difficult information.

**Table 2:** Students’ reasons why they appreciate instructor use of humor in college science classrooms.

Theme	Description of theme	% Responses (n = 1475)	Example student quote	Example student quote
*Humor positively changes the classroom atmosphere*				
Makes class more interesting, fun, or exciting	Student indicates that when instructors use humor in college science classes it makes class more interesting, fun, exciting, entertaining, enjoyable, engaging, or less boring.	49.4%	“When humor is used in class it just makes the time more fun and enjoyable rather than just listening to someone speak for an hour and a half about science.”	“I find that humor helps to make classes more enjoyable in general and that one simple laugh can help put you in the right mood for the rest of the day, which is especially helpful when you're a science major with organic chemistry at 7:30am.”
Lightens the mood of class	Student indicates that when instructors use humor in college science classes it lightens the mood of the class, makes the atmosphere friendlier, more relaxed, more comfortable, more inviting, or less intimidating.	21.8%	“Science is very black and white, and it is nice to lighten the mood of the classroom sometimes.”	“Humor brings an air of lightness into the lecture. Not so heavy.”
Gives students a break from hard content	Student indicates that when instructors use humor in college science classes it gives students a break from difficult science content, allows them time to process the material, or breaks up a lot of information.	7.8%	“When instructors use humor in class, I feel like it gives the students a moment of relief or laughter that is mostly never seen in the dense material covered in science courses.”	"Typically, the information we learn is sometimes hard to understand, so when humor is used, our brains get a brief break to re-group before learning more hard stuff."
*Humor improves students’ experience during class*				
Engages students during class	Student indicates that when instructors use humor in college science classes it changes students' behavior causing them to listen more, pay more attention, be more involved, be more present, be more engaged, or focus on the material.	26.5%	“I appreciate when an instructor uses humor in class because it can help keep students engaged in the topics especially when the class is nearing a close.”	“For me, humor in any class increases my attention level and my willingness to participate in the class. I think it’s more important to do for science class because the material can be very dry and repetitive, so any comedic relief is nice.”
Enhances student learning	Student indicates that when instructors use humor in college science classes they learn more in class or that humor helps students remember, retain, recall, or understand content.	21.4%	“Humor makes points and concepts in class easier to remember/memorize”	“When instructors use humor during any class, it allows me to connect more to the info (…) Maybe I remember a joke or something they said that helps me remember the info.”
Reduces stress-related emotions about class	Student indicates that when instructors use humor in college science classes it causes students to feel more calm or less anxious, nervous, stressed, or tense about learning science content or about the class broadly.	8.5%	“It takes away a bit of the stress that we have when we’re learning something in class that might be difficult for us to understand.”	"Science is one of the harder subjects to be found on a college course list, and with this comes a lot of stress and anxiety, so when a teacher takes the time to joke around, it takes some of the edge off."
*Humor enhances relationships between students and instructors*				
Makes the instructor more relatable or personable	Student indicates that when instructors use humor in college science classes it makes the instructor more relatable, more personable, more human, or the student feels like they have more in common with the instructor.	13.3%	“When my professors use humor, it makes them more relatable. Using humor also makes them more ‘real’ to me.”	“I appreciate when instructors use humor in the classroom because it's a reminder they are people just like us.”
Makes the instructor more approachable	Student indicates that when instructors use humor in college science classes it makes students feel less intimidated, more comfortable, or less nervous approaching the instructor.	7.6%	“By using humor, the instructors seem to be more approachable. Therefore, I am more likely to approach them and ask them questions after class.”	“The professor using humor helps me feel comfortable enough with the professor so that I can ask questions.”
Builds a relationship between the instructor and the student	This category extends beyond relating to or approaching the instructor. Student indicates that the distance between instructor and student is decreasing or indicates that there is a connection or bond being built between the student and instructor.	5.5%	“When a professor is funny or tells a lot of jokes, it helps break down the barriers between students and professors that prevent the two from forming a better relationship.”	"I think that it creates a better relationship between the students and the teacher."

Students also highlighted that humor improves students’ experiences during class. For example, 26.5% of students described that when science instructors use humor, it can cause students to pay more attention in class or to be more engaged with the material and 21.4% of students perceived that humor helps them retain science content and can even enhance their learning. Additionally, students (8.5%) described that science classrooms can cause them to feel stressed or anxious, but instructor use of humor can reduce students’ stress related emotions about the class.

The final overarching category that emerged from the data was that instructor use of humor can enhance the relationship between the instructor and the student. Students (13.3%) described that when science instructors use humor it makes the instructor more personable or relatable and helps students realize that the instructor is a “real person.” In fact, some students (7.6%) perceive that when instructors use humor they appear more approachable and students are more likely to go to them for help or advice. Lastly, students (5.5%) perceived that science instructors’ use of humor can go beyond making the instructors seem more personable and approachable and help build a relationship between instructors and students.

#### Study I Conclusion

Nearly all students (98.8%) appreciate when instructors use humor in college science classrooms. Students appreciate science instructors’ use of humor because it positively changes the classroom atmosphere, improves students’ experience in class, and enhances the relationship between students and the instructor.

### Study II: How do instructors’ use of funny humor, unfunny humor, and offensive humor in college science courses affect student attention to course content, instructor relatability, and student sense of belonging to the course? Are there gender differences in the extent to which students report being affected by funny, unfunny, and offensive humor?

In general, the use of humor has been shown to positively impact students. However, while instructors likely intend for students to find their humor funny, instructors’ use of humor in college science classrooms may not be perceived by all students as funny, and some humor may even be perceived by students as offensive. Yet, no prior study has explored how instructor humor that students perceive to be unfunny or offensive affects students in science courses. Thus, we were interested in exploring the impact of funny, unfunny, and offensive humor on student experiences in class. Further, we tested whether there were gender differences in the extent to which funny, unfunny, and offensive humor impacts student attention to course content, instructor relatability, and student sense of belonging to the science course. We acknowledge that gender identity is not binary (male/female) and recognize that some students identify with non-binary gender identities. Unfortunately, there were too few students who identified as non-binary to include them in the gender analyses in this study.

#### Study II Methods

On the humor survey, students were asked to provide an example of a time that an instructor used humor in a college science course and they thought that it was funny (n = 1637). Then, students were asked how their example of the instructor’s use of funny humor affected their attention to course content, which they answered on a 5-point Likert scale: 1 = It made me pay a lot less attention to course content, 2 = It made me pay a little less attention to course content, 3 = It did not affect my attention to course content, 4 = It made me pay a little more attention to course content, 5 = It made me pay a lot more attention to course content. Students were also asked how their example of the instructor’s use of funny humor influenced instructor relatability, which they answered on a 5-point Likert scale: 1 = It made the instructor a lot less relatable, 2 = It made the instructor a little less relatable, 3 = It did not affect how relatable the instructor was to me, 4 = It made the instructor a little more relatable, 5 = It made the instructor a lot more relatable. Finally, students were asked how their example of the instructor’s funny use of humor affected their sense of belonging to their science class, which they answered using a 5-point Likert scale: 1 = It made me feel like I belonged to the class a lot less, 2 = It made me feel like I belonged to class a little less, 3 = It did not affect my sense of belonging to the class, 4 = It made me feel like I belonged to class a little more, 5 = It made me feel like I belonged to the class a lot more. We designed a 5-point Likert-scale to measure these constructs and included a neutral choice (e.g. It did not affect my attention to course content) because we expected that some humor may not affect a student’s behavior or feelings during class, and when we piloted these questions during think-aloud interviews, we found this to be true.

Next, students were asked to provide an example of a time that an instructor used humor in a college science course and they did *not* find it funny. After students provided the example that they did not think was funny, they were asked whether they perceived the example of instructor humor as offensive (1411 students provided an unfunny example that they did not perceive as offensive (unfunny humor) and 159 students provided an unfunny example that they perceived as offensive (offensive humor)). Then, using the same format of questions described above, students were asked to report how the example of an instructor’s use of humor that they did not find funny affected their attention to course content, instructor relatability, and their sense of belonging to the class. We used multinomial logistic regression to determine whether there were gender differences in the extent to which students reported that funny, unfunny, and offensive humor affected their attention to course content, instructor relatability, and sense of belonging to the course. Multinomial logistic regression is an approach for modeling the relationship between more than two categorically distributed dependent variables- in this case, whether a student reported that a type of humor had a positive impact, no impact, or a negative impact on an outcome variable (student attention to course content, instructor relatability, and sense of belonging to the class) and predictor variables, in this case, student gender. For each type of instructor humor- funny, unfunny, and offensive- we ran three multinomial models to explore the effect of that particular type of instructor humor on students’ reported attention to course content, instructor relatability, and sense of belonging to the class, respectively. Each multinomial model consists of a set of two independent binary logistic regression models. We provide the results of each regression by listing the focus category followed by the reference category and the respective p-value (e.g. focus category/reference category, p-value). There are several ways to interpret model coefficients from logistic regression; the most accessible way is to interpret the natural exponential of the estimated coefficient, which is the factor of change in odds that females compared to males will report that humor affected them in a particular way (e.g. did not affect their sense of belonging vs. increased their sense of belonging), also referred to as the “odds ratio.” The odds ratio can be considered a standardized effect size statistic because the explanatory variable, gender, is binary [51,52].

#### Study II Results

**Fig 2:**
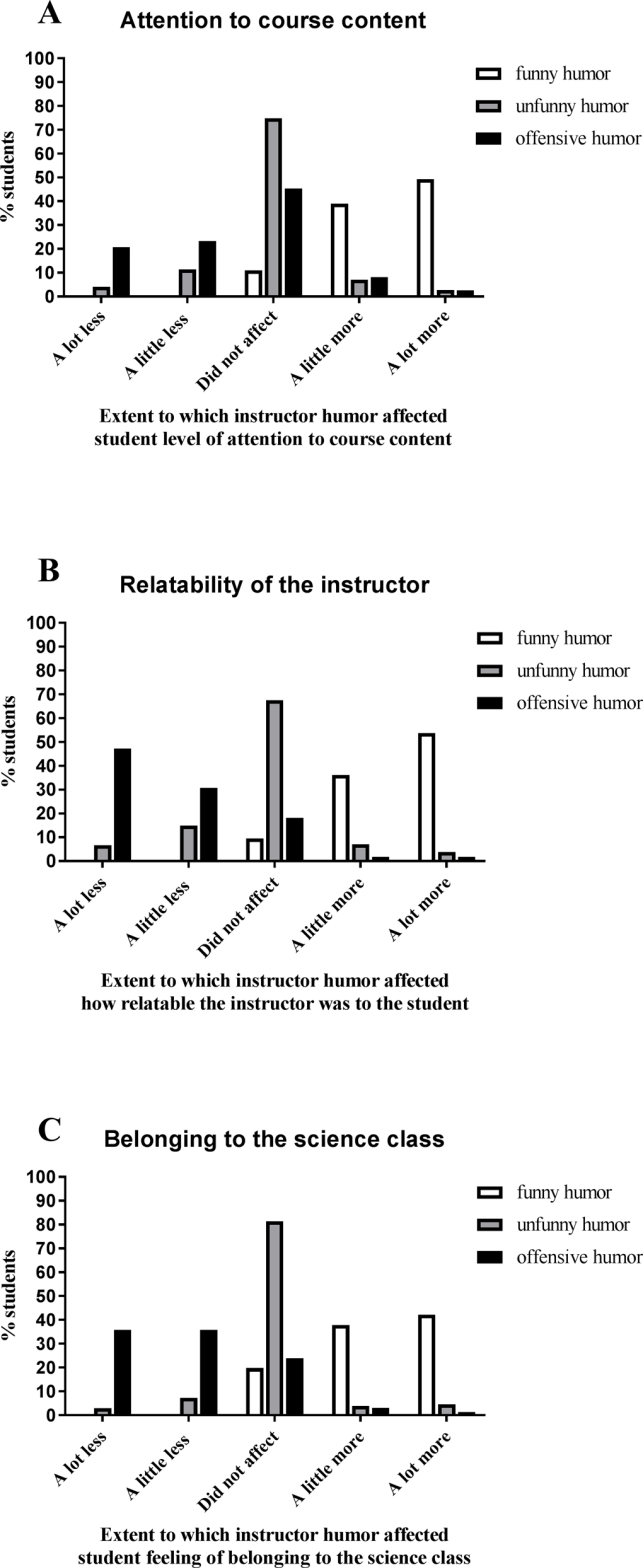
A. Student perception of how instructor use of funny, unfunny, and offensive humor affect their attention to course content. B. Student perception of how funny, unfunny, and offensive humor affect instructor relatability. C. Student perception of how funny, unfunny, and offensive humor affect their sense of belonging to the course.

**Attention to course content.** We found that the majority of students reported that an instructor’s use of funny humor caused them to pay either a little more (39.0%) or a lot more (49.2%) attention to course content. For 11.1% of students, an instructor’s use of funny humor did not affect their attention to course content and for less than 1% of students, it caused them to pay attention to course content less (**Fig 2A**). Females were not significantly more likely than males to report that funny humor makes them pay more attention to course content (more attention/less attention, p = 0.85; more attention/no effect, p = 0.23). All model coefficients, z values, p values, and significant odds ratios are listed in **Table 3**.

**Table 3:** Multinomial regression coefficients for models used to determine whether there are gender differences in the extent to which funny, unfunny, and offensive humor affects student self-reported attention to course content, instructor relatability, and sense of belonging to the class.

		Intercept β±CI (z-value, p-value)	Gender: female (ref:male) β±CI (z-value, p-value)	Standardized effect size- odds ratio that females compared to males will report that humor affected their attention in a specific way^a^
*Dependent variable = Student attention to course content*				
Funny humor (n = 1608)	Increased attention^b^ (ref: No effect)	1.97 ± 0.24 (z = 15.83, p <0.001)	0.19 ± 0.31 (z = 1.20, p = 0.23)	
	Increased attention (ref: Decreased attention)	4.88 ± 0.98 (z = 9.73 p < 0.001)	0.12 ± 1.27 (z = 0.19, p = 0.85)	
Unfunny humor (n = 1380)	No effect (ref: Increased attention)	1.76 ± 0.50 (z = 13.56, p < 0.001)	0.49 ± 0.35 (z = 2.69, p = 0.007)	Females were 1.6x more likely than males to report that unfunny humor has no effect on attention to course content compared to reporting that it increased their attention.
	No effect (ref: Decreased attention)	1.71 ± 0.25 (z = 13.48, p < 0.001)	-0.22 ±0.31 (z = -1.42, p = 0.15)	
Offensive humor (n = 153)	No effect (ref: Increased attention)	1.22 ± 1.00 (z = 2.41, p = 0.02)	0.22 ± 0.78 (z = 0.37, p = 0.71	
	No effect (ref: Decreased attention)	0.06 ± 0.69 (z = 0.17, p = 0.86)	-0.08 ± 0.78 (z = -0.20, p = 0.84)	
*Dependent variable = Instructor relatability*				
Funny humor (n = 1608)	Increased relatability (ref: No effect)	2.11 ± 0.25 (z = 16.08, p < 0.001)	0.22 ± 0.33 (z = 1.31, p = 0.19)	
	Increased relatability (ref: Decreased relatability)	4.90 ± 0.98 (z = 9.76 p = 0.001)	0.81 ± 1.51 (z = 1.07, p = 0.29)	
Unfunny humor (n = 1378)	No effect (ref: Increased relatability)	1.43 ± 0.24 (z = 12.00, p < 0.001)	0.82 ± 0.35 (z = 4.54, p = 0.001)	Females were 2.3x more likely than males to report that unfunny humor has no effect on instructor relatability compared to reporting that it increased instructor relatability.
	No effect (ref: Decreased relatability)	1.31 ± 0.22 (z = 11.50, p < 0.001)	-0.27 ± 0.27 (z = -1.91, p = 0.06)	
Offensive humor (n = 153)	Decreased relatability (ref: Increased relatability)	1.83 ± 1.06 (z = 3.40, p < 0.001)	2.72 ±2.23 (z = 2.39, p = 0.02)	Females were 15.2x more likely than males to report that offensive humor decreased instructor relatability compared to reporting that it increased instructor relatability.
	Decreased relatability (ref: No effect)	1.02 ± 0.76 (z = 2.63, p = 0.01)	0.59 ± 0.90 (z = 1.27, p = 0.20)	
*Dependent variable = Student sense of belonging to the course*				
Funny humor (n = 1607)	Increased sense of belonging (ref: No effect)	1.35 ± 0.20 (z = 13.44, p < 0.001)	0.09 ± 0.25 (z = 0.67 p = 0.50)	
	Increased sense of belonging (ref: Decreased sense of belonging)	11.90 ± 34.38 (z = 0.68, p = 0.50)	-5.19 ± 34.4 (z = -0.29, p = 0.77)	
Unfunny humor (n = 1381)	No effect (ref: Increased sense of belonging)	1.77 ± 0.25 (z = 14.09, p < 0.001)	1.01 ± 0.39 (z = 5.00, p < 0.001	Females were 2.7x more likely than males to report that unfunny humor has no effect on their belonging compared to reporting that it increased their belonging.
	No effect (ref: Decreased sense of belonging)	2.36 ± 0.31 (z = 14.46, p < 0.001)	-0.40 ± 0.39 (z = -2.03, p = 0.04)	Females were 1.5x less likely than males to report that unfunny humor has no effect on their belonging compared to reporting that it decreases their belonging.
Offensive humor (n = 153)	Decreased belonging (ref: Increased belonging)	1.57 ± 0.96 (z = 3.40, p < 0.001)	2.18 ± 1.71 (z = 2.51, p = 0.01)	Females were 8.8x more likely than males to report that offensive humor decreased belonging compared to reporting that it increased their belonging.
	Decreased belonging (ref: no effect)	0.98 ± 0.76 (z = 2.51, p = 0.01)	0.13 ± 0.88 (z = 0.29, p = 0.77)	

^a^The odds ratio that females compared to males will report that a type of humor will affect them in a specific way is reported out for significant findings.

^b^The focus category for each respective analysis is the dependent variable reported by the majority of students.

The majority of students (74.8%) reported than an instructor’s use of unfunny humor did not affect their attention to course content. However, for nearly 16% of students, an instructor’s use of unfunny humor caused them to pay a little less (11.4%) or a lot less (4.1%) attention to content. For some students, even though they found an instructor’s use of humor unfunny, it still caused them to pay attention to the content either a little more (7.1%) or a lot more (2.7%) (**Fig 2A**). Females were 1.6x more likely than males to report that unfunny humor had no effect on their attention compared to reporting that it made them pay more attention (no effect/more attention, p = 0.007). However, there was no significant gender difference in the extent to which students reported that unfunny humor had no effect on their attention when compared to causing them to pay less attention (no effect/less attention, p = 0.15).

For many students, if the instructor’s use of humor was offensive to them, it negatively influenced their attention to course content, as 23.3% of students described that an instructor’s use of offensive humor caused them to pay attention to course content a little less and 20.8% of students described that it caused them to pay attention a lot less. For 45.3% of students, an instructor’s use of offensive humor did not affect their attention to course content. There were some students who, despite finding the instructor’s use of humor offensive, reported that it made them pay attention to course content either a little more (8.2%) or a lot more (2.5%) (**Fig 2A**). Females were no more or less likely than males to report that offensive humor had no effect on their attention to course content (no effect/less attention, p = 0.84, no effect/more attention, p = 0.71).

**Instructor relatability.** On average, an instructor’s use of funny humor in college science classes increased instructor relatability for students. The majority of students reported that an instructor’s use of funny humor made the instructor either a little more relatable (36.2%) or a lot more relatable (53.7%). While 9.5% of students reported that the instructor’s use of funny humor did not affect how relatable the instructor was to the student, only 0.5% of students reported that it made the instructor less relatable to them (**Fig 2B**). Females were not significantly more likely than males to report that funny humor makes the instructor more relatable (more relatable/no effect, p = 0.19, more relatable/less relatable p = 0.29).

For most students (67.5%), an instructor’s use of unfunny humor did not affect how relatable the instructor of the course was to them. However, some students reported that an instructor’s use of unfunny humor made the instructor a little less relatable (14.9%) or a lot less relatable (6.7%). Interestingly, about 10% of students reported that even when they did not find an instructor’s use of humor funny, it still made the instructor seem a little more relatable (7.0%) or a lot more relatable (3.8%) (**Fig 2B**). Females were 2.3x more likely than males to report that unfunny humor had no effect on their instructor’s relatability compared to a positive impact (no effect/more relatable, p = 0.001). However, there was no significant gender difference in the extent to which students reported that unfunny humor had no effect on instructor relatability compared to a negative impact (no effect/less relatable, p = 0.06).

If the instructor’s use of unfunny humor was offensive, the majority of students reported that it made the instructor a little less (30.8%) or a lot less (47.2%) relatable. For 18.2% of students, the instructor’s offensive humor did not affect how relatable the instructor was for the student, and a minority of students (3.8%) reported that although they perceived the instructor’s humor as offensive, it made the instructor more relatable to the student (**Fig 2B**). Females were 15.2x more likely than males to report that offensive humor made the instructor of the course less relatable compared to more relatable (less relatable/more relatable, p = 0.02). However, there was no significant gender difference in the extent to which students reported that offensive humor made the instructor of the course less relatable compared to having no effect on instructor relatability (less relatable/no effect, p = 0.20).

**Sense of belonging.** We were also interested to see how instructor use of humor affects students’ sense of belonging to the course. On average, instructors using funny humor increased students’ sense of belonging to their science class; instructor use of funny humor increased most students’ sense of belonging to the course a little more (37.8%) or a lot more (42.2%). For 19.8% of students, the instructors’ use of funny humor did not affect their sense of belonging and only 0.2% of students reported that the funny example caused them to feel as though they belonged to class less (**Fig 2C**). Females were not significantly more likely than males to report that funny humor makes them feel as though they belong more to the class (belong more/no effect, p = 0.50, belong more/belong less, p = 0.77).

On average, science instructors’ use of unfunny humor did not seem to influence students’ sense of belonging to their science class. The majority of students (81.4%) reported that instructors’ use of unfunny humor did not affect their sense of belonging. There was no clear trend for how instructors’ use of unfunny humor affected the remaining students; less than 10% of students reported that an instructor’s use of unfunny humor caused them to feel like they belonged to class a little more (3.9%) or a lot more (4.6%) and approximately 10% of students reported that the instructor’s use of unfunny humor caused them to feel like they belonged to class a little less (7.3%) or a lot less (2.9%) (**Fig 2C**). Females were 2.7x more likely than males to report that unfunny instructor humor has no effect on their belonging compared to a positive impact (no effect/belong more, p < 0.001). However, females were 1.5x less likely than males to report that unfunny humor has no effect on their belonging compared to a negative impact (no effect/belong less, p = 0.04).

When students perceived the instructors’ use of unfunny humor to be offensive, it was more likely to negatively affect their sense of belonging. While 23.9% of students reported that an instructor’s use of offensive humor did not affect their sense of belonging to the course, 35.8% of students reported that it made them feel like they belonged to the class a little less and 35.8% of students reported that it made them feel like they belonged to the class a lot less (**Fig 2C**). Females were 8.8x more likely than males to report that offensive humor caused them to feel as though they belong less to the course compared to reporting that offensive humor made them feel as though they belong more to the course (belong less/belong more, p = 0.01). However, there was no significant gender difference in the extent to which students reported that offensive humor made them feel as though they belong less to the course when compared to reporting that offensive humor had no effect on their sense of belonging (belong less/no effect, p = 0.77).

#### Study II Conclusion

Instructors’ use of humor that students found funny positively affected the majority of students’ attention to course content, instructor relatability, and students’ sense of belonging to the course. Instructors’ use of humor that students did not find funny did not have an impact on most students’ attention to course content, instructor relatability, or students’ sense of belonging to the class. However, if students considered an instructor’s unfunny example of humor to be offensive, for most students, it negatively influenced their sense of belonging to the course and the instructor’s relatability. For most students, offensive humor either did not have an effect on their attention to course content or caused them to pay less attention to course content.

There were few gender differences in how funny, unfunny, and offensive humor affected student-reported attention to course content, instructor relatability, and sense of belonging to the course. This suggests that females and males have similar reactions to humor that they find funny and that they have similar reactions to humor that they find offensive. The differences that were observed indicated that females were more likely than males to report that unfunny humor did not affect them compared to reporting that it had a positive effect on their attention, instructor relatability, or sense of belonging. This isn't necessarily surprising because very few students reported that unfunny instructor humor affected them positively and these students were mostly male. Similarly, female students were more likely than males to report that offensive humor had a negative impact on their attention and instructor relatability compared to reporting a positive impact. Once again, very few students reported that offensive humor positively affected them and those who did were mostly male.

### Study III: When instructors use humor in college science classes, what potentially humorous subjects are students likely to find funny? What potentially humorous subjects are students likely to find offensive? Are there potentially humorous subjects that male or female students are more likely to find funny or offensive?

Given the positive impact of funny instructor humor on students in science classrooms and the negative impact of offensive humor on students in science classrooms, it would be helpful to know what potentially humorous subjects students are most likely to find funny and offensive if joked about by an instructor in the context of a college science course.

#### Study III Methods

To identify common potentially humorous subjects, 16 researchers interviewed a convenience sample of 95 college students about the last funny joke that they heard and the last offensive joke that they heard. These were not necessarily jokes told by an instructor in class, but jokes that the student had heard most recently. Two researchers (KMC and SEB) reviewed all 190 examples (95 examples of funny humor and 95 examples of offensive humor), recorded the subject of each joke, and created a list of unique subjects that were mentioned by at least three college students. The interviews of college students took place in February 2017, shortly after the 2017 United States presidential inauguration, which was reflected in the subjects that were recorded. We chose to include all subjects even if they were specific to a particular time or event. We also noted that some of the subjects reported were phrased as broader categories (e.g. religious individuals), so we added more specific subsets of these categories (e.g. Mormons, Christians, Catholics, Jewish people, Muslims).

We included the list of 34 potentially humorous subjects on the humor survey that was sent out to students in college science courses. On the humor survey, students were presented with the list of 34 potentially humorous subjects and asked “If a college science instructor were to tell a joke in class, which of the following jokes might you find funny? Please select all that you might find funny.” For the next question, students were presented with the same list of 34 potentially humorous subjects and asked “If a college science instructor were to tell a joke in class, which of the following jokes might you find offensive? Please select all that you might find offensive.” The question explicitly asked students about “jokes” but the responses were phrased with a focus on the joke subject (e.g. jokes about dogs, jokes about politics).

Given prior research that shows that females and males can interpret humor differently [25,26], we were interested in exploring whether there were differences in the subjects that females and males find funny and offensive when joked about by an instructor in the context of a college science course. We used logistic regression to determine whether there were gender differences in what subjects students reported that they might find funny and offensive. Logistic regression is an approach for modeling the relationship between a dependent variable with two categories, such as whether a student perceives a subject to be funny or not- and an explanatory variable, such as gender. Because there were 34 comparisons for subjects that students might find funny, and 34 comparisons for subjects students might find offensive, we applied the Bonferroni correction for significance at the p < 0.05 level for each set of comparisons. The Bonferroni-adjusted p-value needed for significance is p < 0.001. All logistic regression coefficients are listed in the Supplemental Information (S1 and S2 Tables).

#### Study III Results

The potentially humorous subjects that emerged from student interviews could be categorized as subjects related to United States politics (6 subjects: politics, Republicans, Democrats, Donald Trump (the 45^th^ President of the United States), Hillary Clinton (the 67^th^ United States Secretary of State and the Democratic Party’s nominee for the President of the United States in 2016), Sean Spicer (served as the White House Press Secretary in 2017)), subjects related to sex or bodily functions (3 subjects: sex, genitalia, farts/poop), subjects related to entertainment (2 subjects: television, sports), subjects related to relationships (2 subjects: relationships, divorce), subjects related to college (2 subjects: college, students), subjects related to animals (3 subjects: cute animals, dogs, cats), and subjects related to social identities (14 subjects: old people, women, weight, Mormons, Christians, Catholics, Mexicans, Immigration/Immigrants, Jewish people, African Americans, gay or lesbian people, Muslims, transgender people, people with disabilities). Social identities are based on group memberships and provide individuals with a sense of who they are. Two subjects could not be organized into a larger category: science and food puns (**Table 4).**

**Table 4:** The percent of students who, if a science instructor were to tell a joke about a specific subject, might find the joke funny and might find the joke offensive.

Potentially humorous subjects	% students who might find jokes about subject funny if told by a science instructor	% students who might find jokes about subject offensive if told by a science instructor
**Science**	**89.3%**	**1.5%**
**College**	**84.7%**	**1.5%**
**Television**	**75.9%**	**1.3%**
Food puns	67.3%	1.5%
Relationships	62.3%	8.8%
Cute animals	55.9%	3.6%
Dogs	55.7%	4.5%
Cats	53.2%	3.4%
Sports	51.7%	4.0%
Students	51.5%	16.3%
Politics	48.5%	16.4%
Donald Trump	45.9%	17.2%
Sex	43.9%	18.9%
Farts or poop	33.3%	11.4%
Hillary Clinton	27.5%	23.3%
Old people	27.3%	29.6%
Genitalia	23.4%	33.8%
Republicans	23.2%	35.2%
Divorce	21.6%	28.2%
Sean Spicer	20.8%	13.9%
Democrats	20.6%	39.7%
Women	16.2%	61.6%
Weight	15.8%	48.1%
Mormons	15.5%	45.2%
Christians	15.0%	51.1%
Catholics	12.9%	49.5%
Mexicans	12.2%	60.6%
Immigration/Immigrants	12.0%	49.4%
Jewish people	11.2%	57.1%
African Americans	10.8%	60.9%
Gay or lesbian people	10.4%	58.8%
Muslims	10.1%	62.4%
Transgender people	10.0%	59.9%
People with disabilities	8.2%	63.7%

The table is organized by subjects that the largest percent of students might find funny to subjects that the smallest percent of students might find funny. Subjects that the majority of students might find funny are highlighted in light grey. Subjects that the majority of students might find offensive, which are all subjects related to social identities, are highlighted in dark grey. Subjects that at least 75% of students find funny and that may be considered relatively inoffensive because less than 2% of students reported that they might find the subject offensive, are bolded.

At least half of the students surveyed reported that, if a science instructor told a joke, they might find the joke funny if it were about science (89.3%), college (84.7%), television (75.9%), food puns (67.3%), relationships (62.3%), cute animals (55.9%), dogs (55.7%), cats (53.2%), sports (51.7%), and students (51.5%) **(Table 4)**. Subjects that at least half of the students reported that they might be offended by are all social identities: people with disabilities (63.7%), Muslims (62.4%), women (61.6%), African Americans (60.9%), Mexicans (60.6%), transgender people (59.9%), gay or lesbian people (58.8%), Jewish people (57.1%), and Christians (51.1%) **(Table 4)**. There were three subjects that appeared to be perceived of as universally funny, yet inoffensive because at least three quarters of students reported that they might find the subject funny and less than 2% of students reported that they might find the subject offensive: science (89.3% find funny, 1.5% find offensive), college (84.7% find funny, 1.5% find offensive), and television (75.9% find funny, 1.3% find offensive) **(Table 4)**.

We found that, in general, males were more likely to report that they find jokes about the subjects funny, while females were more likely to report that they find jokes about the subjects offensive. There were 23 subjects that males were more likely than females to report that they might find funny, including all 14 subjects related to social identities. However, there was only one subject, food puns, that females were more likely than males to report that they might find funny (**Table 5**). Conversely, there were 25 subjects that females were more likely than males to report that they might find offensive, including all 14 subjects related to social identities, all six subjects related to politics, and both subjects related to relationships. Males were never more likely than females to report that they might find a subject offensive (**Table 6**).

**Table 5:** Gender differences in what subjects students report they might find funny if an instructor of a college science course were to tell a joke about them.

Potentially humorous subject	% of females who might find jokes about subject funny if told by a science instructor (n = 1004)	% of males who might find jokes about subject funny if told by a science instructor (n = 606)	Gender of students significantly more likely to find subject funny	p-value^a^	Standardized effect size- odds ratio that males will perceive the subject funny
Science	89.1%	89.6%		0.772	
College	85.5%	83.3%		0.252	
Television	78.7%	71.9%		0.002	
Food puns	71.9%	59.6%	Females	<0.001	1.7x less likely
Relationships	60.7%	65.3%		0.060	
Cute animals	58.6%	51.5%		0.006	
Dogs	58.6%	50.3%		0.001	
Cats	55.2%	49.7%		0.032	
Sports	45.6%	62.0%	Males	<0.001	2.0x more likely
Students	49.2%	54.8%		0.030	
Politics	40.5%	62.0%	Males	<0.001	2.4x more likely
Donald Trump	43.1%	50.7%		0.003	
Sex	39.2%	51.5%	Males	<0.001	1.6x more likely
Farts or poop	31.6%	36.0%		0.070	
Hillary Clinton	19.8%	39.9%	Males	<0.001	2.7x more likely
Old people	21.1%	37.3%	Males	<0.001	2.2x more likely
Genitalia	16.5%	34.3%	Males	<0.001	2.6x more likely
Republicans	16.7%	33.3%	Males	<0.001	2.5x more likely
Divorce	16.0%	30.2%	Males	<0.001	2.3x more likely
Sean Spicer	14.5%	30.7%	Males	<0.001	2.6x more likely
Democrats	12.6%	33.3%	Males	<0.001	3.5x more likely
Women	8.1%	29.4%	Males	<0.001	4.8x more likely
Weight	7.8%	28.5%	Males	<0.001	4.8x more likely
Mormons	9.3%	25.2%	Males	<0.001	3.3x more likely
Christians	8.5%	25.2%	Males	<0.001	3.7x more likely
Catholics	6.7%	22.8%	Males	<0.001	4.1x more likely
Mexicans	5.8%	22.3%	Males	<0.001	4.7x more likely
Immigration/Immigrants	4.9%	23.3%	Males	<0.001	5.9x more likely
Jewish people	4.6%	21.8%	Males	<0.001	5.8x more likely
African Americans	4.5%	20.6%	Males	<0.001	5.5x more likely
Gay or lesbian people	4.0%	20.6%	Males	<0.001	6.2x more likely
Muslims	3.5%	20.5%	Males	<0.001	7.1x more likely
Transgender people	3.6%	19.8%	Males	<0.001	6.6x more likely
People with disabilities	2.7%	16.8%	Males	<0.001	7.3x more likely

The odds ratio that males compared to females might perceive the subject funny are reported for subjects where the gender difference is significant.

^a^A Bonferroni-adjusted alpha level of <0.001 was used.

**Table 6:** Gender differences in what subjects students report they might find offensive if an instructor of a college science course were to tell a joke about them.

Potentially humorous subject	% of females who might find jokes about subject offensive if told by a science instructor (n = 1004)	% of males who might find jokes about subject offensive if told by a science instructor (n = 606)	Gender of students significantly more likely to find subject offensive	p-value^a^	Standardized effect size- odds ratio that females will perceive the subject offensive
Science	1.2%	1.8%	-	0.31	
College	1.6%	1.5%	-	0.87	
Television	1.1%	1.8%	-	0.23	
Food puns	1.0%	2.3%	-	0.04	
Relationships	10.8%	5.8%	Females	<0.001	2.0x more likely
Cute animals	4.0%	3.1%	-	0.38	
Dogs	5.2%	3.5%	-	0.11	
Cats	4.0%	2.6%	-	0.16	
Sports	5.0%	2.5%	-	0.02	
Students	20.0%	10.6%	Females	<0.001	2.1x more likely
Politics	20.9%	8.7%	Females	<0.001	2.8x more likely
Donald Trump	21.3%	10.9%	Females	<0.001	2.2x more likely
Sex	24.4%	10.2%	Females	<0.001	2.8x more likely
Farts or poop	13.1%	8.9%	-	0.01	
Hillary Clinton	30.8%	10.4%	Females	<0.001	3.5x more likely
Old people	36.9%	18.0%	Females	<0.001	2.7x more likely
Genitalia	43.5%	18.2%	Females	<0.001	3.5x more likely
Republicans	44.1%	21.1%	Females	<0.001	2.9x more likely
Divorce	34.2%	18.8%	Females	<0.001	2.2x more likely
Sean Spicer	17.1%	8.9%	Females	<0.001	2.1x more likely
Democrats	50.7%	22.3%	Females	<0.001	3.6x more likely
Women	76.8%	37.3%	Females	<0.001	5.5x more likely
Weight	61.8%	26.4%	Females	<0.001	4.5x more likely
Mormons	55.5%	29.2%	Females	<0.001	3.0x more likely
Christians	61.3%	36.0%	Females	<0.001	2.8x more likely
Catholics	61.3%	31.4%	Females	<0.001	3.5x more likely
Mexicans	71.6%	43.4%	Females	<0.001	3.3x more likely
Immigration/Immigrants	61.6%	30.0%	Females	<0.001	3.7x more likely
Jewish people	68.1%	39.6%	Females	<0.001	3.3x more likely
African Americans	73.2%	41.6%	Females	<0.001	3.8x more likely
Gay or lesbian people	71.5%	38.4%	Females	<0.001	4.0x more likely
Muslims	73.7%	44.7%	Females	<0.001	3.5x more likely
Transgender people	73.2%	38.4%	Females	<0.001	4.4x more likely
People with disabilities	77.6%	41.4%	Females	<0.001	4.9x more likely

The odds ratio that females compared to males might perceive the subject offensive are reported for subjects where the gender difference is significant.

^a^A Bonferroni-adjusted alpha level of <0.001 was used.

#### Study III Conclusion

In college science classrooms, students are most likely to find instructor jokes funny if they are about college, science, or television and students are most likely to be offended by instructor jokes about social identities, particularly social identities that are historically or currently marginalized in the United States. There are gender differences in whether students might find jokes about specific subjects funny and offensive. Males are more likely to find jokes about social identities funny, while females are more likely to find jokes about social identities offensive.

## Discussion

Despite the potential for humor to positively influence students in science courses, there has been little research on students’ perceptions of science instructor use of humor in the college science classroom. In this manuscript, we document student perceptions of instructor use of humor in college science classrooms, which give insights into how science instructors can use humor to maximize student experiences, while minimizing the potentially negative effects of humor.

Overwhelmingly, students reported that they appreciated when instructors used humor. However, this was the first study to explore why instructor use of humor may be particularly appreciated in college science courses. Students acknowledged that science courses can be stressful and that science content is especially difficult, but that humor helps lighten the mood of science classes, decreases stress levels, and improves students’ perceived ability to remember science content. Future studies could explore the extent to which humor benefits students in science courses compared to courses with more positive reputations such humanities classes [3].

For the majority of students in this study, when science instructors used humor that students did not think was funny, it did not have an effect on their attention to course content, how relatable they perceived the instructor to be, or their sense of belonging to the class. Thus, if an instructor tells a joke that falls flat, it is likely not harming students. However, this is not the case if students find an instructor’s use of humor to be offensive. We found that if students perceive a science instructor’s use of humor as offensive, it can negatively influence how relatable students perceive the instructor to be. Previous research also suggests that negative and hostile humor can harm student-instructor relationships, particularly if students previously perceived the instructor to be immediate, or physically and psychologically close with students, because the negative humor contradicts their warm and open style [53]. Further, we found that instructors’ use of offensive humor tends to decrease student sense of belonging to the course, which has been shown to be an important predictor of student retention [54,55]. Over 40% of students reported that offensive humor can also decrease their attention to course content. Offensive humor may negatively affect student attention because it increases student cognitive load, or the amount of information that a student can hold in their working memory. This may be particularly true for students if the joke is offensive because it targeted a social identity group that they belong to [56–58].

Notably, if a college science instructor is able to tell a joke that males and females think is funny, our findings suggest that both genders benefit equally. Similarly, if a college science instructor tells a joke that males and females both perceive as offensive, there is little evidence to suggest that females would be more harmed than male students. Therefore, based on our findings, females are more likely to be negatively affected by humor because they find more subjects offensive, not because of their response to the offensive humor.

Our study identified three subjects- science, college, and television- that the vast majority (> 75%) of students found funny, and that a small minority (<2%) of students found offensive. Neither males nor females were more likely to find these subjects funny or offensive. Thus, we conclude that instructors may want to consider these subjects when integrating humor into the college science classroom. Incorporating jokes about science into the classroom may be particularly beneficial to instructors because prior literature suggests that jokes about course content may be received positively by students, even when delivered by instructors who students consider less immediate, or more psychologically distant [59]. Further, jokes about science may be helpful to include in class if an instructor is using humor to promote student learning gains. Researchers have started to investigate whether the subject of humor matters for student learning and have found that humor illustrating course concepts can improve student learning in the course compared to humor that is unrelated to course content [60–62]. However, there are different ways to tell a joke about science, including ways to make it offensive, so instructors will want to be thoughtful in how they deliver jokes about science.

It is important to note that the subject of a joke is not enough to definitively determine whether the joke will be perceived as funny. Who is telling the joke, how the joke is delivered, other subjects within the joke, and the audience member’s culture and sense of humor all influence how the joke will be received [16,47,63]. Future research should explore the relative influence of these parameters in order to identify ways to maximize the benefits of instructor use of humor and minimize the negative consequences. Finally, we only explored differences between men and women in their perceptions of instructor use of humor, but future work could extend to exploring how other social identities differentially perceive instructor humor and the relative impact of instructor humor on students in science.

### Limitations

This research was conducted across multiple classes at one institution in the Southwestern United States. Humor can be highly dependent on culture and thus, these findings may not be applicable to non-Western cultures [9,47]. This research was dependent on student self-report of their perceptions of instructor humor and how that humor may impact them, which could be influenced by the extent to which a student has previously experienced instructor humor. We asked students what subjects they might find funny and offensive if a science instructor were to tell a joke about them. There was no way to control for what type of instructor the student imagined would be telling the joke or the possible context of the jokes that students might have thought about. Further, although we sampled from multiple science courses, biology majors were overrepresented in our sample, which could have biased our results. However, we know of no literature suggesting that students from different science majors would interpret humor differently and students were asked to think broadly about their science courses, which for a typical biology major would include biology, physics, and chemistry courses. Thus, generalizations from this study should be made with caution and these findings would benefit from being replicated at different types of institutions in other countries and across the United States.

### Conclusions

The majority of students appreciate when instructors use humor in college science classrooms. While funny instructor humor tended to positively affect student attention to course content, instructor relatability, and student sense of belonging to the course, for most students, unfunny humor did not seem to affect these constructs. Students reported that offensive instructor humor tended to decrease their sense of belonging to the course and instructor relatability. There were few significant gender differences in how funny instructor humor and offensive instructor humor affected students, but numerous significant gender differences in the topics that students found funny and offensive. Lastly, students are most likely to find a joke funny and least likely to find a joke offensive if the joke is about science, television, or college and students are most likely to find instructor jokes offensive if they are about social identities.

## Supporting information

S1 Table Results of logistic regression to explore gender differences in what subjects students find funny. (DOCX)

S2 Table Results of logistic regression to explore gender differences in what subjects students find offensive. (DOCX)

S1 File Questions from the final humor survey. (DOCX)

S2 File Coding rubric for student reasons why they appreciate when instructors use humor in college science classrooms. (DOCX)

S3 File Anonymized data. (XLSX)
