# Supplementary material for: To be funny or not to be funny: Gender differences in student perceptions of instructor humor in college science courses
Source: PLoS One. 2018 Aug 15;13(8):e0201258. doi: 10.1371/journal.pone.0201258 (PMC6093647; doi:10.1371/journal.pone.0201258)
Supplement: S1 Table — (DOCX) [file pone.0201258.s001.docx]

**S1 Table. Results of logistic regression to explore gender differences in what subjects students find funny.**

| **Potentially humorous subjects** | **Intercept**  β±CI (z value, p value) | **Gender: male (ref:female)**  β±CI (z value, p value) |
| --- | --- | --- |
| Science | 2.11 ± 0.20  (z = 20.75, p < 0.001) | 0.05 ± 0.33  (z = 0.29, p = 0.772) |
|  |  |  |
| College | 1.77 ± 0.09  (z = 19.78, p < 0.001) | -0.16 ± 0.27  (z = -1.15, p = 0.252) |
| Television | 1.31 ± 0.16  (z = 16.95, p < 0.001) | -0.36 ± 0.24  (z = -3.07, p = 0.002) |
| Food puns | 0.94 ± 0.14  (z = 13.39, p < 0.001) | -0.55 ± 0.22  (z = -5.09, p < 0.001) |
| Relationships | 0.43 ± 0.12  (z = 6.70, p < 0.001) | 0.20 ± 0.21  (z = 1.88, p = 0.060) |
| Cute animals | 0.34 ± 0.12  (z = 5.40, p < 0.001) | -0.29 ± 0.20  (z = -2.77, p = 0.006) |
| Dogs | 0.34 ± 0.12  (z = 5.40, p < 0.001) | -0.33 ± 0.20  (z = -3.22, p = 0.001) |
| Cats | 0.21 ± 0.12  (z = 3.28, p = 0.001) | -0.22 ± 0.20  (z = -2.15, p = 0.032) |
| Sports | -0.18 ± 0.12  (z = -2.77, p = 0.006) | 0.67 ± 0.20  (z = 6.36, p < 0.001) |
| Students | -0.03 ± 0.12  (z = -0.51, p = 0.614) | 0.22 ± 0.20  (z = 2.17, p = 0.030) |
| Politics | -0.38 ± 0.12  (z = -5.96, p < 0.001) | 0.87 ± 0.22  (z = 8.29, p <0.001) |
| Donald Trump | -0.28 ± 0.12  (z = -4.34, p < 0.001) | 0.30 ± 0.20  (z =2.94, p = 0.003) |
| Sex | -0.44 ± 0.12  (z = -6.76, p < 0.001) | 0.50 ± 0.20  (z = 4.78, p < 0.001) |
| Farts or poop | -0.77 ± 0.14  (z = -11.39, p < 0.001) | 0.20 ± 0.22  (z = 1.82, p = 0.070) |
| Hillary Clinton | -1.40 ± 0.16  (z = -17.65, p < 0.001) | 0.99 ± 0.22  (z = 8.63, p < 0.001) |
| Old people | -1.32 ± 0.16  (z = -17.04, p < 0.001) | 0.80 ± 0.22  (z = 6.99, p < 0.001) |
| Genitalia | -1.61 ± 0.16  (z = -19.06, p < 0.001) | 0.97 ± 0.24  (z = 8.05, p < 0.001) |
| Republicans | -1.60 ± 0.16  (z = -18.98, p = < 0.001) | 0.91 + 0.24  (z = 7.55, p < 0.001) |
| Divorce | -1.67 ± 0.18  (z = -19.25, p < 0.001) | 0.82 ± 0.24  (z = 6.63, p < 0.001) |
| Sean Spicer | -1.77 ± 0.18  (z = -19.78, p < 0.001) | 0.96 ± 0.25  (z = 7.62, p < 0.001) |
| Democrats | -1.93 ± 0.18  (z = -20.35, p < 0.001) | 1.24 ± 0.25  (z = 9.67, p < 0.001) |
| Women | -2.43 ± 0.24  (z = -21.00, p < 0.001) | 1.56 ± 0.29  (z = 10.64, p < 0.01) |
| Weight | -2.47 ± 0.24  (z = -20.98, p < 0.001) | 1.56 ± 0.29  (z = 10.5, p < 0.001) |
| Mormons | -2.28 ± 0.22  (z = -20.96, p < 0.001) | 1.20 ± 0.27  (z = 8.34, p < 0.001) |
| Christians | -2.38 ± 0.22  (z = -21.00, p < 0.001) | 1.30 ± 0.29  (z = 8.81, p < 0.001) |
| Catholics | -2.64 ± 0.25  (z = -20.86, p < 0.001) | 1.41 ± 0.31  (z = 8.89, p < 0.001) |
| Mexicans | -2.79 ± 0.27  (z = -20.64, p < 0.001) | 1.54 ± 0.33  (z = 9.25, p < 0.001) |
| Immigration/Immigrants | -2.97 ± 0.29  (z = -20.28, p < 0.001) | 1.78 ± 0.35  (z = 10.14, p < 0.001) |
| Jewish people | -3.04 ± 0.29  (z = -20.14, p < 0.001) | 1.76 ± 0.35  (z = 9.76, p < 0.001) |
| African Americans | -3.06 ± 0.29  (z = -20.06, p < 0.001) | 1.71 ± 0.35  (z = 9.38, p < 0.001) |
| Gay or lesbian people | -3.18 ± 0.31  (z = -19.72, p < 0.001) | 1.83 ± 0.37  (z = 9.65, p < 0.001) |
| Muslims | -3.32± 0.33  (z = -19.30, p < 0.001) | 1.96 ± 0.39  (z = 9.85, p < 0.001) |
| Transgender people | -3.29 ± 0.33  (z = -19.39, p < 0.001) | 1.89 ± 0.39  (z = 9.56, p < 0.001) |
| People with disabilities | -3.59 ± 0.39  (z = -18.40, p < 0.001) | 1.99 ± 0.43  (z = 8.92, p < 0.001) |
