# Supplementary material for: To be funny or not to be funny: Gender differences in student perceptions of instructor humor in college science courses
Source: PLoS One. 2018 Aug 15;13(8):e0201258. doi: 10.1371/journal.pone.0201258 (PMC6093647; doi:10.1371/journal.pone.0201258)
Supplement: S2 Table — (DOCX) [file pone.0201258.s002.docx]

**S2 Table. Results of logistic regression to explore gender differences in what subjects students find offensive.**

| **Potentially humorous subjects** | **Intercept**  β±CI (z value, p value) | **Gender: female (ref:male)**  β±CI  (z value, p value) |
| --- | --- | --- |
| Science | -3.99 ± 0.59  (z = -13.12, p < 0.001) | -0.42 ± 0.82  (z = -1.01, p = 0.31) |
|  |  |  |
| College | -4.19 ± 0.65  (z = -12.49, p < 0.001) | 0.07 ± 0.82  (z = 0.17, p = 0.87) |
| Television | -3.99 ± 0.59  (z = -13.12, p < 0.001) | -0.51 ± 0.82  (z = -1.19, p = 0.23) |
| Food puns | -3.74 ± 0.53  (z = -13.85, p < 0.001) | -0.85 ± 0.82  (z = -2.05, p = 0.04) |
| Relationships | -2.79 ± 0.33  (z = -16.03, p < 0.001) | 0.67 ± 0.39  (z = 3.35, p < 0.001) |
| Cute animals | -3.43 ± 0.45  (z = -14.72, p < 0.001) | 0.24 ± 0.55  (z = 0.88, p = 0.38) |
| Dogs | -3.32 ± 0.43  (z = -14.98, p < 0.001) | 0.42 ± 0.51  (z = 1.59, p = 0.11) |
| Cats | -3.60 ± 0.49  (z = -14.24, p < 0.001) | 0.42 ± 0.59  (z = 1.42, p = 0.16) |
| Sports | -3.67 ± 0.51  (z = -14.05, p < 0.001) | 0.73 ± 0.59  (z = 2.43, p = 0.02) |
| Students | -2.14 ± 0.25  (z = -16.16, p < 0.001) | 0.75 ± 0.29  (z = 4.88, p < 0.001) |
| Politics | -2.34 ± 0.27  (z = -16.31, p < 0.001) | 1.02 ± 0.31  (z = 6.21, p < 0.001) |
| Donald Trump | -2.10 ± 0.25  (z = -16.12, p < 0.001) | 0.80 ± 0.29  (z = 5.25, p < 0.001) |
| Sex | -2.17 ± 0.25  (z = -16.20, p < 0.001) | 1.04 ± 0.29  (z = 6.81, p < 0.001) |
| Farts or poop | -2.32 ± 0.27  (z = -16.30, p < 0.001) | 0.44 ± 0.33  (z = 2.56, p = 0.01) |
| Hillary Clinton | -2.05 ± 0.25  (z = -16.05, p < 0.001) | 1.24 ± 0.29  (z = 8.56, p < 0.001) |
| Old people | -1.52 ± 0.22  (z = -14.35, p < 0.001) | 0.98 ± 0.29  (z = 7.87, p < 0.001) |
| Genitalia | -1.51 ± 0.22  (z = -14.29, p < 0.001) | 1.24 ± 0.24  (z = 10.12, p < 0.001) |
| Republicans | -1.32 ± 0.20  (z = -13.23, p < 0.001) | 1.08 ± 0.24  (z = 9.16, p < 0.001) |
| Divorce | -1.46 ± 0.20  (z = -14.06, p < 0.001) | 0.81 ± 0.24  (z = 6.53, p < 0.001) |
| Sean Spicer | -2.32 ± 0.27  (z = -16.30, p < 0.001) | 0.75 ± 0.33  (z = 4.53, p < 0.001) |
| Democrats | -1.24 ± 0.20  (z = -12.80, p < 0.001) | 1.28 ± 0.24  (z = 10.99, p < 0.001) |
| Women | -0.52 ± 0.16  (z = -6.18, p < 0.001) | 1.71 ± 0.22  (z = 15.26, p < 0.001) |
| Weight | -1.02 ± 0.18  (z = -11.12, p < 0.001) | 1.50 ± 0.22  (z = 13.34, p < 0.001) |
| Mormons | -0.89 ± 0.18  (z = -9.91, p < 0.001) | 1.11 ± 0.22  (z = 10.09, p < 0.001) |
| Christians | -0.58 ± 0.16  (z = -6.81, p < 0.001) | 1.03 ± 0.22  (z = 9.71, p < 0.001) |
| Catholics | -0.78 ± 0.18  (z = -8.95, p < 0.001) | 1.24 ± 0.22  (z = 11.40, p < 0.001) |
| Mexicans | -0.27 ± 0.16  (z = -3.24, p = 0.001) | 1.19 ± 0.22  (z = 11.05, p < 0.001) |
| Immigration/Immigrants | -0.85 ± 0.18  (z = -9.54, p < 0.001) | 1.32 ± 0.22  (z = 11.99, p < 0.001) |
| Jewish people | -0.42 ± 0.16  (z = -5.08, p < 0.001) | 1.18 ± 0.22  (z = 11.99, p < 0.001) |
| African Americans | -0.33 ± 0.16  (z = -4.12 p < 0.001) | 1.34 ± 0.22  (z = 12.35, p < 0.001) |
| Gay or lesbian people | -0.47 ± 0.16  (z = -5.64 p < 0.001) | 1.39 ± 0.22  (z = 12.77, p < 0.001) |
| Muslims | -0.21 ± 0.16  (z = -2.60 p = 0.009) | 1.24 ± 0.22  (z = 11.43, p < 0.001) |
| Transgender people | -0.47 ± 0.16  (z = -5.64 p < 0.001) | 1.48 ± 0.22  (z = 13.44, p < 0.001) |
| People with disabilities | -0.35 ± 0.16  (z = -4.20 p < 0.001) | 1.59 ± 0.22  (z = 14.19, p < 0.001) |
