# Supplementary material for: To be funny or not to be funny: Gender differences in student perceptions of instructor humor in college science courses
Source: PLoS One. 2018 Aug 15;13(8):e0201258. doi: 10.1371/journal.pone.0201258 (PMC6093647; doi:10.1371/journal.pone.0201258)
Supplement: S1 File — (DOCX) [file pone.0201258.s003.docx]

**Questions from the final humor survey**

Please indicate the degree to which you agree with the following statement: I appreciate when instructors use humor in college science classrooms.

- Strongly agree
- Agree
- Slightly agree
- Slightly disagree
- Disagree
- Strongly disagree

*If student selects strongly agree or agree:*

Please explain your reasoning for why you appreciate when instructors use humor in college science classes.

*All students:*

In 3-4 sentences, please describe a time when an instructor used humor in a college science class and you thought it was funny.

Please describe why you thought the example you gave was funny.

How did the instructor's use of funny humor that you gave in the example affect your sense of belonging to the class?

- It made me feel like I belonged to the class a lot more
- It made me feel like I belonged to the class a little more
- It did not affect my sense of belonging to the class
- It made me feel like I belonged to the class a little less
- It made me feel like I belonged to the class a lot less

How did the instructor’s use of funny humor that you gave in the example affect how relatable the instructor was to you?

- It made the instructor a lot more relatable to me
- It made the instructor a little more relatable to me
- It did not affect how relatable the instructor was to me
- It made the instructor a little less relatable to me
- It made the instructor a lot less relatable to me

How did the instructor's use of funny humor you gave in the example affect your attention to course content?

- It made me pay a lot more attention to the course content
- It made me pay a little more attention to the course content
- It did not affect my level of attention to the course content
- It made me pay a little less attention to the course content
- It made me pay a lot less attention to the course content

In 3-4 sentences, please describe a time when an instructor used humor in a college science class and you thought it was **not** funny.

Please describe why you thought the example you gave was not funny.

Was the unfunny example of an instructor using humor that you just gave offensive to you?

- Yes
- No

How did the instructor's use of unfunny humor that you gave in the example affect your sense of belonging to the class?

- It made me feel like I belonged to the class a lot more
- It made me feel like I belonged to the class a little more
- It did not affect my sense of belonging to the class
- It made me feel like I belonged to the class a little less
- It made me feel like I belonged to the class a lot less

How did the instructor’s use of unfunny humor that you gave in the example affect how relatable the instructor was to you?

- It made the instructor a lot more relatable to me
- It made the instructor a little more relatable to me
- It did not affect how relatable the instructor was to me
- It made the instructor a little less relatable to me
- It made the instructor a lot less relatable to me

How did the instructor's use of unfunny humor you gave in the example affect your attention to course content?

- It made me pay a lot more attention to the course content
- It made me pay a little more attention to the course content
- It did not affect my level of attention to the course content
- It made me pay a little less attention to the course content
- It made me pay a lot less attention to the course content

We are interested in learning more about what students find funny and offensive in college science classrooms. Consider a situation where an instructor of your college science class tells a joke. We would like you to indicate what types of jokes **you** find funny and what types of jokes **you** find offensive. We recognize that there can be a wide range of jokes within a particular subject (e.g. jokes about sports) so you may find this subject both funny and offensive depending on the context.  If this is the case, just mark the subject (e.g. jokes about sports) under both scenarios. Please only indicate subjects that you personally find funny or find offensive, not subjects that you think others might find funny or be offended by.

If a college science instructor were to tell a joke in class, which of the following jokes might **you** find funny? Please select all that you might find **funny.**

- jokes about science
- jokes about women
- jokes about Christians
- jokes about TV
- jokes about Mexicans
- jokes about Democrats
- jokes about genitalia
- jokes about Muslims
- jokes about Donald Trump
- jokes about cats
- jokes about African Americans
- jokes about Catholics
- jokes about students
- jokes about sex
- jokes about cute animals
- jokes about Mormons
- jokes about Republicans
- jokes about gay or lesbian people
- jokes about politics
- jokes about farts or poop
- jokes about college
- jokes about relationships
- jokes about divorce
- jokes about sports
- jokes about weight
- jokes about transgender people
- jokes about dogs
- jokes about old people
- jokes about immigration
- jokes about people with disabilities
- food puns
- jokes about Jewish people
- jokes about Sean Spicer
- jokes about Hillary Clinton

If a college instructor were to tell a joke in class, which of the following jokes might **you** find offensive? Please select all that you might find **offensive**.

- jokes about science
- jokes about women
- jokes about Christians
- jokes about TV
- jokes about Mexicans
- jokes about Democrats
- jokes about genitalia
- jokes about Muslims
- jokes about Donald Trump
- jokes about cats
- jokes about African Americans
- jokes about Catholics
- jokes about students
- jokes about sex
- jokes about cute animals
- jokes about Mormons
- jokes about Republicans
- jokes about gay or lesbian people
- jokes about politics
- jokes about farts or poop
- jokes about college
- jokes about relationships
- jokes about divorce
- jokes about sports
- jokes about weight
- jokes about transgender people
- jokes about dogs
- jokes about old people
- jokes about immigration
- jokes about people with disabilities
- food puns
- jokes about Jewish people
- jokes about Sean Spicer
- jokes about Hillary Clinton

I most closely identify as a

- Biological Sciences major
- Chemistry or Biochemistry major
- Physics major
- Geosciences major
- Other Major, please describe
- Decline to state

I most closely identify as

- Female
- Male
- Other, please describe
- Decline to state

I most closely identify as

- American Indian, Native American, or Alaskan Native
- Asian
- Black or African American
- Hispanic or Latino or Spanish
- Native Hawaiian or Other Pacific Islander
- White/Caucasian
- Other, please describe
- Decline to state

How old are you?
