# Supplementary material for: To be funny or not to be funny: Gender differences in student perceptions of instructor humor in college science courses
Source: PLoS One. 2018 Aug 15;13(8):e0201258. doi: 10.1371/journal.pone.0201258 (PMC6093647; doi:10.1371/journal.pone.0201258)
Supplement: S2 File — (DOCX) [file pone.0201258.s004.docx]

**Coding rubric for student reasons why they appreciate when instructors use humor in college science classrooms.**

| **Theme** | **Description of Theme** |
| --- | --- |
| Humor positively changes the classroom atmosphere | |
| Makes class more interesting, fun, or exciting | Student indicates that when instructors use humor in college science classes it makes class more interesting, fun, exciting, entertaining, enjoyable, or less boring. If the student indicates that the use of humor makes the class more engaging, captures or grabs attention of the students but did not mention how it affects students’ behavior (e.g. students become more engaged), the statement is coded under this theme. |
| Lightens the mood of class | Student indicates that when instructors use humor in college science classes it lightens the mood or makes the class friendlier, more comfortable, or less intimidating. Student indicates the use of humor makes class more inviting or relaxed. If the student says that the class or material feels less stressful, the statement is coded under this theme because it is referencing the class environment. |
| Gives students a break from hard content | Student indicates that when instructors use humor in college science classes it gives them a break from the content, allows them time to process, or it breaks up a lot of information. |
| Humor improves students’ experience during class | |
| Engages student during class | Student indicates that when instructors use humor in college science classes it changes students' behavior causing them to listen more, pay more attention, be more involved, be more present or engaged, or causes them to focus. |
| Enhances student learning | Student indicates that when instructors use humor in college science classes it helps students remember, retain, recall, or understand content, or it helps students learn more in the class. |
| Reduces stress-related emotions about class | Student indicates that when instructors use humor in college science classes it causes students to feel less anxious, nervous, stressed, or tense about learning content or about the class broadly, or it makes them feel calmer. |
| Humor enhances relationships between students and instructors | |
| Makes instructor more relatable or personable | Student indicates that when instructors use humor in college science classes it makes the instructor more relatable, more human, or they feel like they have more in common with the instructor or the instructor is more personable. Category includes when a student states that humor indicates that the instructor does not take themselves too seriously. |
| Makes instructor more approachable | Student indicates that when instructors use humor in college science classes it makes students feel less intimidated, more comfortable, or less nervous approaching instructors. |
| Builds a relationship between the instructor and the student | Student indicates that when instructors use humor in college science classes it builds a relationship or connection between students and instructors. |
